# Not Just a Fish Killer: Multi-Organ Toxicity and Mechanisms of 6PPD-Quinone

**DOI:** 10.3390/toxics14040288

**Published:** 2026-03-28

**Authors:** Pinzhi Dong, Meijun Liu, Haiyan Wang, Jin Chen, Xiaorong Xu, Hailong Su, Ming Qin, Junmin Luo

**Affiliations:** 1 Special Key Laboratory of Gene Detection & Therapy, Key Laboratory of Cancer Prevention and Treatment of Guizhou Province, Department of Immunology, School of Basic Medicine, Center of Immunomolecular Engineering, Innovation & Practice Base for Graduate Students Education, Zunyi Medical University, Zunyi 563000, China; dpzwyyx66258@163.com (P.D.);; 2 Department of Epidemiology and Health Statistics, Zunyi Medical University, Zunyi 563000, China; 3 Biology and Medicine Program, School of Bioengineering, Zhuhai Campus, Zunyi Medical University, Zhuhai 519000, China; 4 Key Laboratory of Basic Pharmacology of Ministry of Education and Joint International Research Laboratory of Ethnomedicine of Ministry of Education, Zunyi Medical University, Zunyi 563000, China

**Keywords:** ecotoxicology, environmental toxicity, tire derivatives, 6PPD-Q, multi-organ toxicity

## Abstract

6PPD-Quinone (6PPD-Q) is a tire derivative formed by the oxidation of N-(1,3-dimethylbutyl)-N′-phenyl-p-phenylenediamine (6PPD), a commonly used antioxidant and ozone stabilizer in rubber products, and has emerged as a significant environmental concern in recent years. It is widely present in the atmosphere, surface lakes, and soil. The primary routes of exposure to 6PPD-Q are the digestive tract and respiratory tract. Studies indicate that it is a major factor causing acute mortality in coastal coho salmon (*Oncorhynchus kisutch*). Reports indicate that 6PPD-Q exhibits greater chemical stability and stronger biological toxicity than 6PPD, demonstrating toxic effects across multiple species. 6PPD-Q has been detected in human urine samples, indicating a need for heightened attention to its potential health risks. 6PPD-Q exhibits multi-organ toxicity in organisms, including intestinal, hepatic, neurotoxic, and reproductive toxicity. Its potential toxic mechanisms are associated with oxidative stress and inflammatory responses, and it can disrupt amino acid metabolism, carbohydrate metabolism, and lipid metabolism while interfering with signal transduction pathways by binding to specific receptors. This paper reviews the environmental contamination of 6PPD-Q, explores its potential toxic effects on organisms and underlying mechanisms, analyzes gaps in the current research and future trends, and contributes to a better understanding of its environmental occurrence and biological hazards.

## 1. Background

The pollution caused by automobiles has always been a major concern. N-(1,3-Dimethylbutyl)-N′-phenyl-p-phenylenediamine (6PPD) is a commonly used additive in tires and various rubber products. It functions as an anti-aging agent and ozone stabilizer, extending the service life of rubber goods such as tires [1]. 6PPD can be released into the environment from tire wear microplastic particles (TWMPs), where it reacts with atmospheric ozone to form the more chemically stable compound 6PPD-quinone (6PPD-Q), which is then further dispersed throughout the environment via runoff and atmospheric transport [1,2,3,4]. In recent years, a phenomenon known as Urban Runoff Mortality Syndrome has emerged in coastal cities across the United States. During the spawning season, coho salmon (*Oncorhynchus kisutch*) returning to urban runoff experience unexplained mass mortality following heavy rainfall. After systematically screening various chemicals in the runoff, the research team confirmed that 6PPD-Q is the primary factor causing Urban Runoff Mortality Syndrome [4,5]. Meanwhile, numerous studies have proven that 6PPD-Q exerts toxic effects across multiple species, with toxicity affecting many organs [6,7,8,9]. These findings warrant urgent attention to the health risks associated with 6PPD-Q. Therefore, this review systematically summarizes the current research status of 6PPD-Q, with particular focus on the following aspects: (1) environmental occurrence and evidence of organismal exposure to 6PPD-Q; (2) multi-species, multi-organ toxicological effects of 6PPD-Q; (3) potential toxicity mechanisms of 6PPD-Q, particularly oxidative stress, inflammation, and metabolic disorders; (4) limitations of existing 6PPD-Q research and future research prospects.

### 1.1. Environmental Distribution of 6PPD-Q

**Figure 1:**
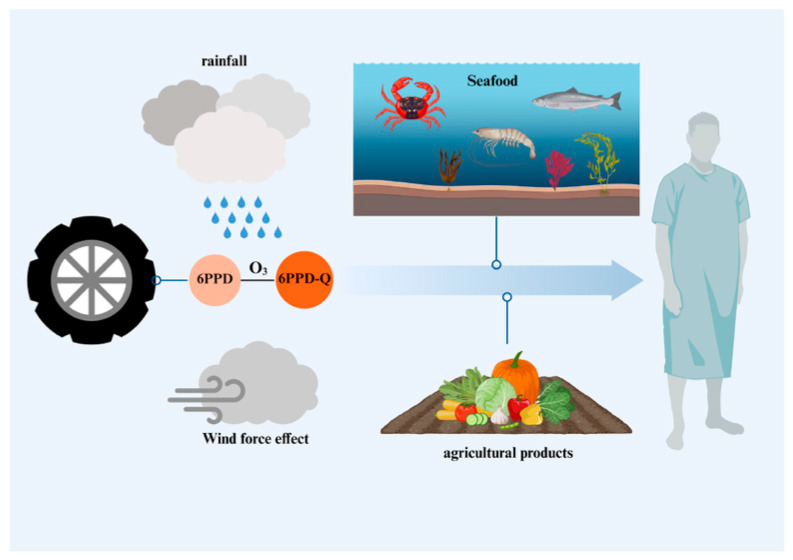
Environmental Exposure to 6PPD-Q. 6PPD in tires can further react with ozone in the environment to form 6PPD-Q, which is then dispersed throughout the environment via rainfall and wind currents. It accumulates in various organisms and ultimately enters the human body either directly or indirectly. Created in BioRender. Pinzhi, D. (2026) https://BioRender.com/jv44t2u, accessed on 22 March 2026.

As an emerging pollutant, the presence of 6PPD-Q has been documented in numerous studies across various environmental media. It has been detected in surface water, atmosphere, soil, and even certain aquatic and agricultural products [10,11,12,13,14,15] (Figure 1). The research team detected 6PPD-Q in the Don River in the Toronto area of Canada, with concentrations of 6PPD-Q in the river peaking at 2.58 µg/L following rainfall [11]. Multiple compounds have been detected in the city water systems of the Pearl River Delta region of South China, with 6PPD-Q concentrated at up to 1562 ng/L [16]. Beyond urban runoff, 6PPD-Q is also present in lakes, estuaries, coastal areas, snow melt, and even tap water [17,18,19,20,21]. Zhuxia et al. [22] sampled dust and soil from electronic waste recycling areas. Results showed that 6PPD-Q was detected in both emerging and traditional recycling areas, with its concentration correlating with temperature.

The tire-related compounds 6PPD-Q and 4-aminodiphenylamine (4-ADPA) were detected in ambient air particulate matter along motorways in the U.S. state of Mississippi, with estimated concentrations of 2.90 and 1.14 ng/L, respectively [12]. Thirty-seven tire-related compounds have been detected in the air of towns and villages in the German state of Saxony, with a median concentration of 4.1 pg/m^3^ for 6PPD-Q [13]. 6PPD-Q is commonly detected in both indoor and outdoor particulate matter in urban areas, exhibiting a distinct diurnal pattern with peak concentrations occurring during daytime hours [23]. Jiawen et al. [14] combined Quick, Easy, Cheap, Effective, Rugged, and Safe (QuEChERS) with high-performance liquid chromatography–tandem mass spectrometry (HPLC/MS-MS), optimized the method, and tested honey and various fish products. Results showed no detection of 6PPD-Q in honey samples, but 6PPD-Q was detected in fish products. Vegetable samples from 9 Swiss retail stores showed varying degrees of 6PPD-Q contamination across 100 vegetable varieties [15]. The above research indicates that the environmental pollution caused by 6PPD-Q should not be overlooked.

More seriously, 6PPD-Q has been confirmed to exist in the human body. Children in the electronic waste area had significantly higher concentrations of 6PPD-Q in their urine compared to children in non-electronic-waste areas [24]. Among 100 volunteers tested in Tianjin, China, over half (86%) had 6PPD-Q detected in their urine, while the detection rate in blood was relatively low (<15%) [25]. 6PPD-Q is also present in human breast milk [26]. These studies suggest an urgent need to investigate exposure to 6PPD-Q, elucidate its potential pathogenic mechanisms, and establish corresponding health threshold standards.

### 1.2. In Vivo Distribution, Metabolism, and Excretion of 6PPD-Q

The widespread presence of 6PPD-Q increases the risk of exposure for living organisms. Following exposure to 6PPD-Q, it exhibits a tendency to accumulate in multiple organs within the body. After zebrafish exposure to 6PPD-Q, the compound primarily accumulates in the brain, intestines, and eyes, with lower levels observed in the liver [27]. In mice administered 6PPD-Q via oral gavage, the compound reached peak systemic concentrations after 1 h of exposure and was primarily distributed throughout multiple organs including adipose tissue, brain, liver, kidney, lung, testis, and heart [28,29]. The strong accumulation of 6PPD-Q in adipose and brain tissues indicates its lipophilic chemical properties, which may be closely associated with the neurotoxicity induced by 6PPD-Q.

The metabolism of 6PPD-Q in vivo primarily occurs in the liver and is closely associated with cytochrome P450 enzymes. The use of cytochrome P450 inhibitors significantly reduces the metabolic rate of 6PPD-Q [30,31,32]. The biotransformation rate of 6PPD-Q exhibits significant interspecies variation. Semi-quantitative analysis indicates differing biotransformation rates among four species: carp, rainbow trout, rats, and humans. Carp demonstrate the fastest conversion rate, while humans exhibit the slowest [33]. This may be related to differences in catalytic enzymes between species [33]. Additionally, the types of metabolites produced by 6PPD-Q also exhibit interspecies differences. 6PPD-Q undergoes metabolism in human and rat liver microsomes to yield seven metabolites [30]. 6PPD-Q can be converted into twelve transformation products in zebrafish embryos, which differ from those in other fish species [7,34]. Fifteen metabolites were detected in mice following exposure to 6PPD-Q [33]. Two metabolites of 6PPD-Q were detected in human urine, while three metabolites were detected in umbilical cord blood [33,35]. Although the types of 6PPD-Q metabolites differ among species, existing research on the metabolic conversion of 6PPD-Q in animals indicates that monooxygenation products and glucuronic acid conjugates are the primary metabolites of 6PPD-Q in vivo [30,33,34].

The primary excretion pathways for 6PPD-Q include fecal excretion and urinary excretion. Metabolites of 6PPD-Q have been detected in the biliary tracts of multiple fish species and excreted via the digestive tract [7]. Both 6PPD-Q and its hydroxylated metabolites have been detected in mouse feces, indicating that 6PPD-Q can be excreted through feces [29,33]. 6PPD-Q and its metabolites have been detected in both mouse and human urine, indicating that urine serves as a significant route for the excretion of 6PPD-Q and its metabolites [28,29,33,36]. Apart from fecal and urinary excretion pathways, there are currently no published reports indicating whether 6PPD-Q can be eliminated from the body through other routes such as sweat, respiration, or tears.

## 2. Toxicity Effects of 6PPD-Q

There are significant interspecies differences in the toxicity of 6PPD-Q to organisms. Its potent toxicity to fish species such as coho salmon has been confirmed [5,37,38]. However, studies indicate that certain freshwater invertebrates exhibit lower sensitivity to 6PPD-Q than salmonid species [39]. For instance, species such as *Hexagenia* spp. and *Planorbella pilsbryi* did not show significant lethal effects following exposure to 6PPD-Q [39]. Some fish species also exhibit tolerance to 6PPD-Q [7]. Exposure to 6PPD-Q at environmentally relevant concentrations shortens the lifespan of *Caenorhabditis elegans* [6,40]. The causes and mechanisms underlying interspecies differences in 6PPD-Q toxicity remain inconclusive, with speculation suggesting that they may be related to variations in catalytic enzymes among different species [33]. Currently, research on the lethal dose of 6PPD-Q for terrestrial vertebrates remains insufficient, necessitating further in-depth studies to explore potential factors influencing interspecies variations in 6PPD-Q toxicity.

**Figure 2:**
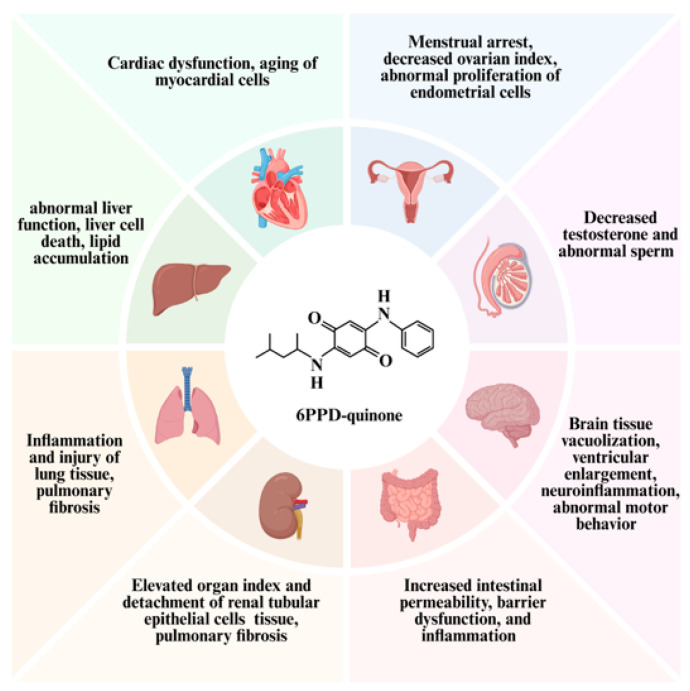
Multi-Organ Toxicity of 6PPD-Q. 6PPD-Q exhibits toxicity to multiple organs including the heart, liver, lungs, kidneys, intestines, brain, and reproductive organs, potentially causing multi-organ damage. Created in BioRender. Pinzhi, D. (2026) https://BioRender.com/udglanx, accessed on 26 March 2026.

Exposure to 6PPD-Q manifests as multi-organ accumulation and multi-organ toxicity (Figure 2). Its effects involve multiple organs and systems including the intestines, liver, nervous system, lungs, testes, and heart [8,27,28].

### 2.1. Intestinal Toxicity of 6PPD-Q

The intestines function as the body’s primary site for digestion and absorption, and they also represent the essential pathway through which orally ingested 6PPD-Q disperses within the body. Multiple studies have shown that exposure to 6PPD-Q can cause intestinal damage in organisms, primarily manifested as structural disruption of the intestinal tract, increased permeability, and heightened expression of inflammatory mediators [41,42,43]. Exposure to 6PPD-Q significantly increased intestinal permeability in *Caenorhabditis elegans*, leading to impaired intestinal barrier function and increased lipofuscin accumulation in intestinal tissues [41,43,44]. In zebrafish models, exposure to 6PPD-Q resulted in pronounced intestinal hypoplasia in juvenile fish [45]. In adult zebrafish exposed to 6PPD-Q, intestinal villi exhibited dose-dependent atrophy, decreased tight junction protein expression, and impaired intestinal barrier function [9,27,45]. Following exposure to 6PPD-Q, mice exhibited marked damage to the jejunum and ileum, with impaired barrier function in both segments [42]. This was characterized by a reduction in goblet cells, thinning and disruption of the intestinal mucosal epithelium, and the loss of crypts, accompanied by inflammatory cell infiltration [8,42]. The levels of inflammatory factors, including Tumor Necrosis Factor-α (TNF-α), Interleukin-6 (IL-6), and Interleukin-1 (IL-1), are upregulated [8,42].

### 2.2. Effects of 6PPD-Q on Gut Microbiota

The gut microbiota is highly diverse and plays an irreplaceable role in maintaining host health. In recent years, multiple studies have confirmed that the gut microbiota exerts significant influence across multiple domains, including host digestion and nutrient absorption, immune regulation, neuro-modulation, and metabolic control [46,47,48,49]. Exposure to 6PPD-Q alters the composition of the gut microbiota. 6PPD-Q significantly altered the β-diversity of the gut microbiota of collembola and significantly reduced the relative abundances of Gammaproteobacteria, Alphaproteobacteria, Actinobacteria, and Holophagae [50]. Following exposure to 6PPD-Q, the composition of various bacterial communities in the gut of zebrafish underwent significant changes, and the relative abundance of *Nocardioides* spp. and *Rhodococcus* spp. increased with prolonged exposure duration [27]. These bacterial genera are involved in the degradation of aromatic hydrocarbons and aliphatic non-methane hydrocarbons, suggesting that they may be involved in the in vivo metabolism of 6PPD-Q [27]. However, there are no published reports on the effects of 6PPD-Q through these two bacterial species on other biological processes. 6PPD-Q altered the abundance of Bacteroidota and Firmicutes in the mouse gut microbiota, increasing Bacteroidota abundance while decreasing Firmicutes abundance [51]. Changes in the gut microbiota inhibit the phosphoinositide 3-kinase (PI3K)/protein kinase B (AKT) signaling pathway [51]. The PI3K/AKT signaling pathway is a classic intracellular signaling pathway that regulates various biological processes in cells and is associated with neuronal regeneration and repair [52,53]. This suggests that 6PPD-Q may cause neuronal damage and cognitive impairment in mice by inducing changes in the gut microbiota and inhibiting the PI3K/AKT signaling pathway [51]. Zhuxia et al. [54] conducted a study on the gut microbiota of children living in an e-waste recycling area and found that within the range of the samples examined, there was a negative correlation between exposure to 6PPD-Q and the α-diversity index of the children’s gut microbiota, suggesting that 6PPD-Q may affect gut microbiota diversity, but more data are needed to support this. These studies suggest that 6PPD-Q can influence gut microbiota distribution, thereby affecting other physiological processes.

### 2.3. Hepatotoxicity of 6PPD-Q

The liver serves as the body’s central hub for material transformation and detoxification, capable of processing a wide range of endogenous and exogenous toxins. 6PPD-Q can cause structural damage and functional abnormalities in the liver. Upon exposure to 6PPD-Q, hepatic function-related indicators including Aspartate Aminotransferase (AST), Alanine Aminotransferase (ALT), Lactate Dehydrogenase (LDH), total bilirubin, and indirect bilirubin exhibited significant alterations, suggesting that 6PPD-Q can induce abnormal liver function [55]. Exposure to 6PPD-Q in zebrafish larvae resulted in abnormal liver development [9]. Adult zebrafish exposed to 6PPD-Q exhibited disorganized hepatocyte cords, cytoplasmic vacuolation in hepatocytes, and an increased number of lipid droplets in liver tissue [9,56]. Mice administered 6PPD-Q via oral gavage exhibited increased liver-to-body weight ratio, elevated AST/ALT ratio, hepatocyte swelling, and increased apoptosis [8,57,58,59]. Yunqing et al. [60] conducted research using a human hepatocyte cell line, demonstrating that 6PPD-Q reduces hepatocyte viability in a dose-dependent manner while increasing reactive oxygen species production in hepatocytes. HepG2 is a human hepatocellular carcinoma cell line commonly used as a model in studies of liver metabolism and toxicol [61,62]. HepG2 cells exhibit abnormal lipid metabolism and increased lipid accumulation following exposure to 6PPD-Q [63]. Regression analysis indicates that serum 6PPD-Q concentrations correlate with liver lesions in outdoor workers [64]. The above studies indicate that 6PPD-Q can induce liver structural and functional lesions by regulating hepatocyte death, oxidative stress, and metabolic abnormalities.

### 2.4. Neurotoxicity of 6PPD-Q

Neurotoxicity is currently the primary focus of research on the biological damage caused by 6PPD-Q. The neurotoxic effects of 6PPD-Q are primarily manifested as anxiety and behavioral abnormalities, neuronal lesions, alterations in brain tissue pathology, and increased permeability [65,66,67]. Zebrafish exposed to 6PPD-Q during the embryonic stage exhibit significant anxiety and reduced social skills upon reaching the juvenile stage; specifically, this manifests as reduced swimming speed, altered body angles, and an increase in both the frequency and duration of attacks by the larvae in the mirror attack assays [66,68]. Adult zebrafish exhibit reduced activity, brain tissue vacuolation, enlarged ventricles, and decreased neurotransmitter secretion [65,66]. 6PPD-Q further increased mortality in rainbow trout, heightened blood–brain barrier permeability, disrupted neurotransmitter expression levels, and ultimately caused motor abnormalities [67]. Following exposure to 6PPD-Q, *Caenorhabditis elegans* exhibited abnormal dopaminergic neuron development, reduced dendritic branching, degeneration of D-type motor neurons, decreased secretion of multiple neurotransmitters including dopamine and Gamma-Aminobutyric Acid (GABA), and impaired motor and sensory perception behaviors [69,70,71,72]. After prolonged exposure to 6PPD-Q, behavioral assessments in mice revealed pronounced cognitive deficits [8,73]. Subsequent studies demonstrated impaired blood–brain barrier integrity, activated microglia, and marked neuroinflammation [8,73]. QuNanet al. [74] found that compared to other organs such as the lungs and cardiovascular system, the neurotoxicity induced by 6PPD-Q is more sustained and irreparable. Mice exposed to 6PPD-Q exhibited long-term anxiety-like behavioral characteristics, with marked neuronal degeneration in the basolateral amygdala of the brain [74]. Additionally, 6PPD-Q may have associations with Parkinson’s disease. The concentration of 6PPD-Q in cerebrospinal fluid differed by a factor of two between Parkinson’s disease patients and unaffected volunteers [75]. Further studies using primary dopaminergic neurons from mice revealed that 6PPD-Q exacerbates the formation of Lewy bodies induced by α-synuclein pre-fibrils [75].

### 2.5. Pulmonary Toxicity of 6PPD-Q

Exposure to 6PPD-Q via inhalation or intraperitoneal injection can cause lung tissue damage, manifesting as alveolar capillary dilation and pulmonary hemorrhage, and promoting the development of pulmonary fibrosis [76,77]. Inhalation of 6PPD-Q in mice causes severe lung tissue damage and the destruction of alveolar macrophages, leading to the reduced secretion of chemokines such as C-C Motif Chemokine Ligand 2 (CCL2) and C-X-C Motif Chemokine Ligand 1 (CXCL1), ultimately exacerbating pneumonia caused by *Klebsiella pneumoniae* [76]. Intraperitoneal injection of 6PPD-Q also causes dilation of pulmonary capillary beds in mouse lung tissue, with substantial exudate appearing in the alveolar spaces accompanied by pulmonary hemorrhage [8]. Long-term repeated injections of 6PPD-Q can also exacerbate inflammatory responses in lung tissue, leading to pulmonary fibrosis changes that impair elastic compliance and increase dead space, thereby severely compromising lung function in mice [77].

### 2.6. Reproductive Toxicity of 6PPD-Q

6PPD-Q can cause damage to the reproductive system, primarily manifested as reduced reproductive capacity, increased germ cell apoptosis, and abnormal sex hormone secretion [78,79,80,81]. Following exposure to 6PPD-Q, *Caenorhabditis elegans* exhibited decreased reproductive capacity, increased germ cell apoptosis, and an abnormal increase in gonadal abnormalities [78,79,82]. Prolonged exposure to 6PPD-Q significantly reduces serum testosterone levels in mice, markedly decreases the proportion of seminiferous tubule sections in stages VII and VIII, causes germ cell detachment from the epithelium, increases apoptosis, reduces total sperm count and motility, and lowers fertilization rates [8,80]. Female mice exposed to 6PPD-Q exhibited menstrual cycle arrest, reduced ovarian index, polycystic-like changes, and granulosa cell damage. Luteinizing hormone levels significantly increased, free testosterone levels rose, and anti-Müllerian hormone levels markedly elevated [81]. Exposure to 6PPD-Q induces abnormal proliferation in human endometrial carcinoma cells and primary human stromal endometrial cells, while reducing the adhesion and growth of trophoblast spheroids on endometrial epithelial cells [83,84].

### 2.7. Cardiac Toxicity of 6PPD-Q

The heart is also a target organ of 6PPD-Q, with its cardiac toxicity primarily manifesting as cardiac dysfunction and myocardial cell senescence [85,86]. Zebrafish embryos exposed to 6PPD-Q exhibited cardiac dysfunction [85]. Mouse cardiomyocyte senescence following 6PPD-Q exposure is characterized by elevated β-galactosidase activity, upregulation of cell cycle inhibitors, induced DNA double-strand breaks, and nuclear lamin B1 remodeling [86].

### 2.8. Nephrotoxicity of 6PPD-Q

6PPD-Q can cause renal injury. Intraperitoneal injection of 6PPD-Q leads to detachment of renal tubular epithelial cells in mice, abnormal cell morphology, nuclear condensation, and frequent hemoglobin casts in the renal medulla [8]. Following intraperitoneal injection of 6PPD-Q in rats, expression levels of inflammatory mediators TNF-α, IL-1β, and IL-6 increased in renal tubules [87]. Markers of tubular injury, Neutrophil Gelatinase-Associated Lipocalin (NGAL) and (Kidney injury molecule 1) Kim-1, were elevated, while apoptosis-related marker Bcl-2-Associated X Protein (BAX) increased and anti-apoptotic marker B-cell Lymphoma-2 (BCL-2) decreased, indicating tubular damage and enhanced apoptosis [87]. However, current research has only examined the renal toxicity of intraperitoneal 6PPD-Q administration. Studies on the effects of exposure via the digestive tract—a significant route—remain absent. Furthermore, the precise mechanism by which 6PPD-Q damages the kidneys remains unclear and warrants further investigation.

## 3. Potential Mechanisms of 6PPD-Q Toxic Effects

### 3.1. Mitochondrial Abnormalities and Oxidative Stress

Oxidative stress refers an imbalance between the production and clearance of reactive oxygen species (ROS) within an organism or cell, leading to the excessive accumulation of ROS and subsequent cellular damage [88]. Excessive ROS can disrupt the normal physiological functions of lipids, proteins, and DNA, impair material metabolism, and is involved in the occurrence and progression of various diseases such as Alzheimer’s disease, atherosclerosis, and diabetes [89,90,91].

Oxidative stress is considered one of the core mechanisms underlying the tissue cytotoxicity of 6PPD-Q. Numerous studies indicate that exposure to 6PPD-Q leads to the accumulation of ROS in cells or in vivo. After absorbing 6PPD-Q added to the soil, Chinese cabbage exhibited significantly elevated ROS levels, along with increased photosynthetic pigment content and antioxidant enzyme activity [92]. Following exposure to 6PPD-Q, ROS levels in the gut of *Caenorhabditis elegans* significantly increased [41,43,44,93]. Following exposure to 6PPD-Q, earthworms exhibited a significant increase in ROS levels within their bodies [94]. Following exposure to 6PPD-Q, intracellular ROS levels in cardiomyocytes significantly increased, accompanied by the upregulation of antioxidant-related genes superoxide dismutase 1 (*sod1*), superoxide dismutase 2 (*sod2*), and nuclear respiratory factor 2 alpha (*nrf2α*) [85,86]. Concurrently, ROS-induced oxidative adducts 8-OHdG formed on DNA guanine bases increased, further precipitating cardiomyocyte senescence [85]. Treatment with the ROS inhibitor N-Acetyl-L-cysteine (NAC) mitigated 6PPD-Q-induced cellular senescence, indicating that oxidative stress is a primary factor in inducing cardiomyocyte senescence [85,86]. Mice exposed to 6PPD-Q exhibited increased accumulation of ROS in vivo [73]. The aforementioned studies indicate that exposure to 6PPD-Q promotes the accumulation of ROS in biological systems, thereby inducing oxidative stress.

**Figure 3:**
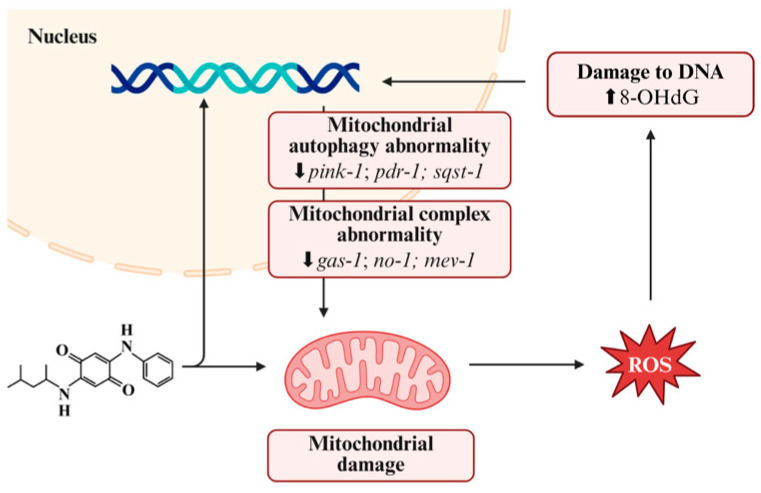
6PPD-Q induces organ damage by triggering oxidative stress. 6PPD-Q can promote ROS accumulation by affecting mitochondrial function and cytochrome P450 activity, leading to oxidative stress. Created in BioRender. Pinzhi, D. (2026) https://BioRender.com/qvuw6tg, accessed on 26 March 2026.

6PPD-Q primarily induces oxidative stress by affecting mitochondrial activity and cytochrome oxidase (Figure 3). Mitochondria serve as one of the primary sources of ROS while also functioning as the cell’s powerhouse and a key site for metabolism. 6PPD-Q accumulates within mitochondria, inhibiting mitochondrial autophagy in *Caenorhabditis elegans* by downregulating the expression of autophagy-related genes pten induced kinase 1 (*pink-1*), Parkinson’s disease related 1 (*pdr-1*), and sequestosome 1 (*sqst-1*) [95]. It increases the accumulation of α-synuclein, reduces the enzymatic activity of mitochondrial complexes [75], and downregulates the genes encoding mitochondrial complex I/II general anesthetic sensitive abnormal (*gas-1*), NADH dehydrogenase [ubiquinone] flavoprotein 1 (*nuo-1*), and succinate dehydrogenase cytochrome b560 subunit (*mev-1*) [40]. This leads to decreased expression of SOD2 and SOD3, promotes ROS accumulation, and exacerbates mitochondrial damage [40]. Additionally, 6PPD-Q can disrupt mitochondrial function by reducing the uptake of the mitochondrial substrate leucine and increasing leucine degradation [96]. These changes ultimately lead to a decrease in mitochondrial membrane potential, increased oxygen consumption, reduced ATP content, and can shorten the lifespan of *Caenorhabditis elegans* [40,95,96,97].

In addition to mitochondrial abnormalities, 6PPD-Q-induced oxidative stress is also associated with cytochrome enzymes, which play a vital role in cellular material metabolism and transformation, participating in the regulation of endoplasmic reticulum stress and ROS production [98,99,100]. Research indicates that cytochrome enzymes mediate the in vivo metabolism of 6PPD-Q, contributing to the increased ROS levels induced by 6PPD-Q exposure [30,32,101].

### 3.2. Inflammatory Response

**Figure 4:**
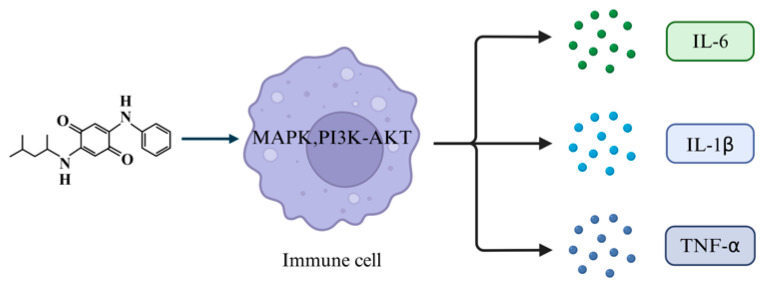
6PPD-Q causes organ damage by inducing inflammation. 6PPD-Q can promote the release of inflammatory mediators by activating immune cells such as macrophages, triggering inflammatory responses that lead to organ damage. Created in BioRender. Pinzhi, D. (2026) https://BioRender.com/4w8i1nm, accessed on 26 March 2026.

Another key mechanism by which 6PPD-Q causes damage to biological tissues and organs is through inflammatory responses. Exposure to 6PPD-Q induces significant inflammatory cell infiltration and the elevated expression of inflammatory cytokines (Figure 4).

Following 6PPD-Q exposure, earthworms exhibited various physiological abnormalities including inflammation and impaired immune function [102]. Following exposure to 6PPD-Q, microglia in mice became activated, with elevated expression levels of the inflammatory factors TNF-α, IL-6, and IL-1β [42,57,73,77]. 6PPD-Q regulates epithelial–mesenchymal transition and inflammatory responses by activating Estrogen Receptor Alpha (ERα) and G Protein-Coupled Estrogen Receptor (GPER), thereby promoting the migration of endometrial cells [83]. Transcriptomic analysis indicates that 6PPD-Q induces the activation of inflammation-related pathways in liver organoids [55]. These studies indicate that the inflammatory response is one of the key mechanisms underlying the biological toxicity of 6PPD-Q.

### 3.3. Metabolic Abnormalities

**Figure 5:**
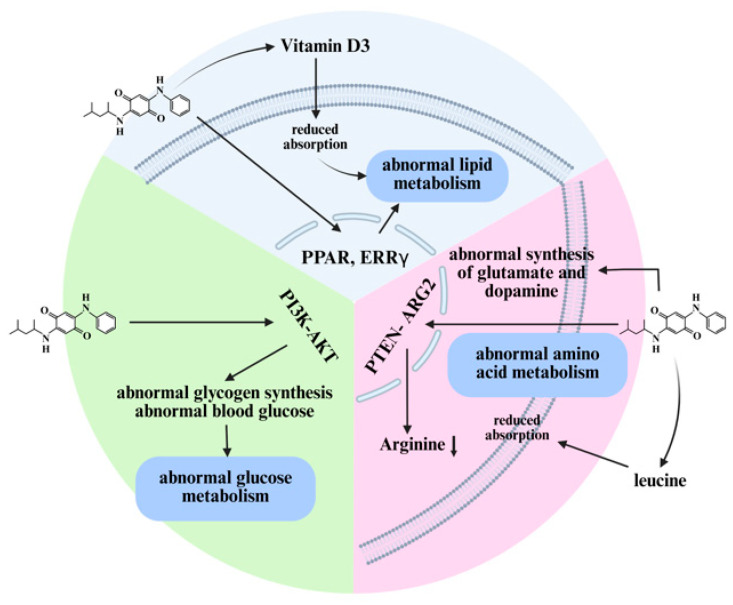
6PPD-Q causes organ damage by inducing metabolic disorders. 6PPD-Q can disrupt lipid metabolism, glucose metabolism, and amino acid metabolism, ultimately leading to organ and systemic damage. Created in BioRender. Pinzhi, D. (2026) https://BioRender.com/6d3c63a, accessed on 26 March 2026.

Multiple studies indicate that 6PPD-Q can affect substance metabolism, with its impact spanning lipid metabolism, glucose metabolism, amino acid metabolism, and other areas [93,96,103] (Figure 5). These metabolic abnormalities are deeply involved in the tissue and organ damage processes induced by 6PPD-Q.

#### 3.3.1. Lipid Metabolism

As one of the three major nutrients, lipids are deeply involved in the composition of cellular structures, participate in signal transduction as signaling molecules, and serve as energy reserves. Disrupted lipid metabolism is associated with the onset and progression of various diseases such as obesity, fatty liver disease, and cardiovascular disorders [104,105,106]. Multiple studies indicate that 6PPD-Q induces lipid metabolism disorders, primarily characterized by increased lipid synthesis and accumulation alongside inhibited fatty acid degradation [94,103]. Following 6PPD-Q exposure, *Caenorhabditis elegans* exhibited markedly increased intestinal lipid accumulation. This lipid accumulation demonstrated transgenerational effects, further leading to lipid accumulation in offspring *Caenorhabditis elegans* [103,107]. An environmental concentration of 6PPD-Q can disrupt the absorption of vitamin D3 by *Caenorhabditis elegans* [108]. Research indicates that vitamin D3 modulates insulin sensitivity, while its metabolites influence fatty acid oxidation and contribute to the development of diseases such as obesity, diabetes, and metabolic syndrome [109,110,111]. This suggests that lipid metabolism disorders induced by 6PPD-Q may be associated with impaired vitamin D3 absorption. Exposure to 6PPD-Q significantly inhibits fatty acid degradation in earthworms [94]. Black-spotted frogs exhibited significantly increased cholesterol and triglyceride levels after exposure to 6PPD-Q [112]. Exposure to 6PPD-Q in mice resulted in multi-organ metabolic disorders affecting the liver, kidneys, spleen, testes, and other organs [56,57,58,113]. In lipid metabolism, this primarily manifested as decreased levels of phosphatidic acid and phosphatidylethanolamine, abnormal glyceride and glycerophospholipid metabolism, and impaired biosynthesis of phosphatidylcholine, phosphatidylethanolamine, and sphingolipids [57,80,113]. These lipids perform diverse biological functions, including cytoskeletal formation, maintenance of membrane structural integrity, and signal transduction [57,80,113]. Lipid metabolism abnormalities induced by 6PPD-Q are associated with Peroxisome Proliferator-Activated Receptor (*PPAR*) and Estrogen-Related Receptor Gamma (*ERRγ*) signaling pathways [63,112]. In the *Caenorhabditis elegans* model, 6PPD-Q-induced lipid metabolism disorders are associated with the metabolic sensors Processed sterol regulatory element binding protein (*SBP-1*) and Mediator of RNA polymerase II transcription subunit 15 (*MDT-15*) [103,107]. The specific mechanism by which 6PPD-Q disrupts lipid metabolism in mammals remains poorly understood, necessitating further investigation into the precise molecular mechanisms underlying its effects on lipid metabolism.

#### 3.3.2. Glucose Metabolism

Carbohydrates are the body’s primary energy source. They can generate ATP through a series of reactions, form glycogen for energy storage, and participate in the synthesis of nucleic acids and nonessential amino acids. 6PPD-Q can cause abnormal glucose metabolism, primarily manifested as abnormal blood glucose levels and impaired glycogen synthesis. By affecting the glycogen synthesis gene glycogen [starch] synthase (*gsy-1*) and reducing the glycogen phosphorylation gene Alpha-1,4 glucan phosphorylase (*pygl-1*), 6PPD-Q increases glycogen accumulation in *Caenorhabditis elegans* [93]. Following the oral administration of 6PPD-Q, mice exhibited increased body weight, elevated fasting blood glucose levels, impaired oral glucose tolerance, and impaired insulin tolerance [114]. Furthermore, the glucose metabolism abnormalities induced by 6PPD-Q are associated with the PI3K-AKT signaling pathway [114].

#### 3.3.3. Amino Acid Metabolism

6PPD-Q disrupts the balance between amino acid absorption and breakdown. 6PPD-Q reduces the expression level of the Large neutral amino acids transporter small subunit 1 (*aat-1*) gene in the gut of *Caenorhabditis elegans*, thereby decreasing leucine absorption in the intestine. It also activates the Branched-chain-amino-acid aminotransferase (*bcat-1*) gene to accelerate leucine catabolism, ultimately leading to a decrease in leucine content within *Caenorhabditis elegans* [96]. Chronic exposure to 6PPD-Q suppresses glutamate synthesis in *Caenorhabditis elegans* and impairs glutamate receptor function [115], disrupting dopamine metabolism and reducing dopamine production [69]. 6PPD-Q also depletes intracellular arginine by acting on the phosphatase and tensin homolog (PTEN)-arginase 2 (ARG2) axis, triggering cellular metabolic reprogramming that promotes the rapid proliferation and migration of cancer cells [116]. Following exposure to 6PPD-Q, the intracellular glutathione content in salmon decreased significantly, a phenomenon associated with oxidative stress [117]. There are currently no published reports on whether 6PPD-Q affects the metabolism of other types of amino acids.

### 3.4. 6PPD-Q Can Bind to Partially Functional Enzymes

6PPD-Q can bind to certain functional enzymes, directly affecting enzyme activity and function. It interacts with LDH through hydrogen bonds and van der Waals forces, altering its conformation and reducing its catalytic activity via non-competitive inhibition [118]. 6PPD-Q can bind to pepsin, altering its secondary structure and inhibiting enzyme activity, which may further lead to issues such as indigestion [119]. These studies indicate that 6PPD-Q can directly bind to and alter enzyme conformation, leading to abnormal enzyme activity and function, thereby influencing disease progression.

## 4. Limitations of Existing 6PPD-Q Studies and Prospects for Future Research

6PPD-Q, as an emerging environmental pollutant that has garnered significant attention in recent years, has been extensively studied for its potential hazards and pathogenic mechanisms. However, given the complex nature of 6PPD-Q’s existence and the intricate metabolic processes it undergoes within living organisms, several significant scientific questions remain to be resolved.

First, it is currently believed that 6PPD-Q primarily originates from the reaction between 6PPD in microplastic particles from tire wear and environmental ozone. However, research remains inconclusive regarding whether other potential sources of 6PPD-Q exist. Furthermore, considering 6PPD’s use as an antioxidant and anti-aging agent in rubber products, its concentration ranges exhibit significant regional variations. Concentrations of 6PPD-Q may be higher in urban areas or industrial zones such as suburban rubber factories. Research on the environmental stability and degradation processes of 6PPD-Q remains limited, with only two studies indicating that its photodegradation pathways in water are influenced by light intensity and temperature [120,121].

Second, exposure through the digestive and respiratory tracts are the primary routes for biological uptake of 6PPD-Q. There are no published reports indicating whether other routes, such as skin exposure, can lead to direct ingestion. Digestive tract intake can be categorized into direct and indirect exposure. Direct exposure occurs through drinking water contaminated with 6PPD-Q or consuming agricultural products contaminated with 6PPD-Q. Indirect exposure occurs via the food chain. However, research on the bioaccumulation and trophic transfer of 6PPD-Q within the food chain remains limited. Research indicates that tire components and their degradation products—6PPD-Q, N,N’-Diphenyl-p-phenylenediamine (DPPD), and N,N’-bis(2-methylphenyl)-1,4-benzenediamine (DTPD)—exhibit strong bioaccumulation potential in estuarine food chains, with a tendency toward trophic magnification [122]. Another study has shown that most tire-derived compounds are bioaccumulative, but it remains unclear whether they are subject to biomagnification [123]. Further research is therefore needed to clarify the accumulation of 6PPD-Q in the food chain.

Third, the metabolic pathways of 6PPD-Q within the body are complex, involving multiple intermediate metabolites. Current detection methods for human exposure to 6PPD-Q are largely limited to the compound itself, while testing for its hydroxylated metabolites and glucuronide metabolites remains insufficient. This limitation affects the accuracy of assessing 6PPD-Q exposure levels. Whether the metabolic intermediates of 6PPD-Q exert toxic effects on tissue cells, and whether the toxicity of 6PPD-Q to various organs and tissues and its mechanisms correlate with the types and proportions of metabolic products, remain unaddressed in the literature. This could serve as a starting point for future in-depth exploration of the toxicity mechanisms of 6PPD-Q.

Fourth, the environmental clearance and in vivo clearance of 6PPD-Q remain to be studied. Research indicates that 6PPD-Q exhibits photochemical degradation characteristics, with factors such as pH, temperature, oxygen concentration, and ultraviolet radiation all influencing the removal of various PPD-Q compounds, including 6PPD-Q [120,121,124]. Previous studies have demonstrated that self-assembled hydrangea-like hollow O,Cl-codoped graphitic phase carbon nitride microspheres, biochar, a multifunctional enzyme system from Trichoderma, and a UV-activated peroxymonosulfate (UV/PMS) system can be employed for the degradation and removal of 6PPD-Q in environmental settings [125,126,127,128]. Currently, there is no effective method for the degradation and clearance of 6PPD-Q in vivo, necessitating further in-depth research into the metabolic transformation patterns of 6PPD-Q within the body.

## 5. Conclusions

In summary, 6PPD-Q is widely present in the environment, and environmental exposure to 6PPD-Q primarily enters the human body via the respiratory and digestive tracts. Once inside the body, 6PPD-Q can cause damage to multiple organs; its toxic effects are linked to the following three mechanisms: (1) 6PPD-Q promotes the accumulation of reactive oxygen species (ROS), inhibits mitochondrial autophagy, interferes with cytochrome enzymes, and induces oxidative stress; (2) 6PPD-Q promotes increased secretion of pro-inflammatory factors such as TNF-α and IL-6, thereby inducing an inflammatory response; (3) 6PPD-Q leads to lipid metabolism disorders by promoting lipid accumulation and inhibiting fatty acid degradation. 6PPD-Q can affect glycogen synthesis, leading to carbohydrate metabolism disorders. 6PPD-Q can also affect the absorption and breakdown of amino acids, leading to amino acid metabolism disorders; (4) 6PPD-Q can bind to enzymes such as LDH, affecting their activity and function.

The risks posed by 6PPD-Q to the environment, living organisms, and humans require urgent attention. The current understanding of 6PPD-Q contamination remains limited. Future research should focus on investigating its environmental transformation and degradation pathways to reduce environmental persistence, assessing human exposure levels and developing corresponding pharmaceuticals to enhance its detoxification and excretion within the body. Effective strategies must be formulated, and appropriate measures implemented to mitigate the risks posed by 6PPD-Q, while simultaneously seeking safer and more effective alternative tire additives.

## Data Availability

No new data were created or analyzed in this study. Data sharing is not applicable to this article.
